# Automatic Detection and Counting of Blood Cells in Smear Images Using RetinaNet

**DOI:** 10.3390/e23111522

**Published:** 2021-11-16

**Authors:** Grzegorz Drałus, Damian Mazur, Anna Czmil

**Affiliations:** Department of Electrical and Computer Engineering Fundamentals, Rzeszow University of Technology, 35-959 Rzeszow, Poland; gregor@prz.edu.pl (G.D.); mazur@prz.edu.pl (D.M.)

**Keywords:** confidence threshold, convolution neural networks, platelet, RBC, WBC

## Abstract

A complete blood count is one of the significant clinical tests that evaluates overall human health and provides relevant information for disease diagnosis. The conventional strategies of blood cell counting include manual counting as well as counting using the hemocytometer and are tedious and time-consuming tasks. This research-based paper proposes an automatic software-based alternative method to count blood cells accurately using the RetinaNet deep learning network, which is used to recognize and classify objects in microscopic images. After training, the network automatically recognizes and counts red blood cells, white blood cells, and platelets. We tested a model trained on smear images and found that the trained model has generalized capabilities. We assessed the quality of detection and cell counting using performance measures, such as accuracy, sensitivity, precision, and F1-score. Moreover, we studied the dependence of the confidence thresholds and the number of learning epochs on the obtained results of recognition and counting. We compared the performance of the proposed approach with those obtained by other authors who dealt with the subject of cell counting and show that object detection and labeling can be an additional advantage in the task of counting objects.

## 1. Introduction

A complete blood count (CBC) is a typical clinical test that provides relevant information for disease diagnosis. The main three types of blood cells are: Red Blood Cells (RBCs), also called erythrocytes, White Blood Cells (WBCs), also called leukocytes, and platelets, also called thrombocytes. CBC provides information about the production of all blood cells, identifies the patient’s ability to carry oxygen by evaluating RBC counts, and allows for immune system evaluation by assessing WBC counts with differential. This test helps diagnose anemia, certain cancers, infections, and many many others, as well as monitor the side effects of certain medications [1]. For this reason, medical laboratories are flooded with a large number of blood and tissue samples that need to be analyzed as accurately as possible and in the shortest possible time. The ability to accurately quantitate specific populations of cells is important for precision diagnostics in laboratory medicine. Thus, medical staff work under heavy loads and time pressure. Medical workers often have to work overtime to analyze all samples on time, causing even greater fatigue of the staff, which may result in mistakes and lower work efficiency [2]. These errors may lead to severe and even fatal consequences in the treatment of patients.

An alternative to traditional manual counting of various cells by specialists are semi-automatic and automatic methods. Automatic detection and counting of cells in images is a difficult and complex task, especially in reality the resolution of input medical images could be very high, at the same time the target cells could easily be extremely dense. Moreover, there are a large number of them in the image, the cells are often overlapped and there are problems with distinguishing cells. This is the principal motivation of automatic cell counting.

There are generally two main approaches in the automated counting of blood cells. We can distinguish traditional methods, which involve several steps such as preprocessing, segmentation, feature extraction, and classification, while other methods are based on deep neural networks (DNN). The selected traditional automatic RBCs counting methods are presented in [3,4]. Various methods of automatic WBC counting are presented in [5,6,7,8,9,10]. Despite the numerous advantages of the automated methods, they also have disadvantages, such as the accuracy of counting and the preparation of cell images. Reliable and accurate cell detection is usually a difficult problem due to a great variability of cells and the complexity of data. Detection can determine the presence of a specific cell in a microscopic image, e.g., lymphocytes. Moreover, detection can be also combined with their counting and quantitative analysis of cells [11]. Automatic cell counting involves obtaining the number of cells in a medical image [12].

In recent years, due to the rapid development of deep learning networks, they have become a key component of many computer vision applications such as object detection, classification or segmentation. The efficiency and efficacy of deep learning in the medical imaging field is unquestionable, as evidenced by a large number of independent studies in different modalities and applications, including those suggested for automatic cell counting [13]. For example, deep learning models that classify various types of erythrocytes were proposed in [14,15]. Vogado et al. [16] proposed LeukNet, which is based on a convolutional neural network (CNN). Acevedo et al. proposed recognition of peripheral blood cell images using CNNs [17]. Automatic white blood cell classification using deep learning models was also presented in [18,19,20,21,22,23]. Automatic identification and counting of all three types of blood cells simultaneously using DNN was proposed in [24].

A literature review indicates that there are only a few articles on the detection and counting of RBCs, WBCs, and platelets simultaneously using deep learning methods [24]. However, it is not clear how to determine the optimal number of epochs and the optimal threshold to achieve the highest performance. We also noted that the obtained results are usually compared only based on accuracy, which in no doubt is an important metric to consider, but it does not always give the full picture. Obtained results should also be discussed in the light of important quality metrics in medical testing: recall, precision, and F1-score. Many works concern recognizing cells in small images that contain just a few cells in the image, while microscopic images can include hundreds of crowded and overlapped cells. Motivated by the lack of a thorough examination of the above issues, we decided to propose our own solution.

This paper aims at developing a precise and automatic method for counting various types of cells in one image using the developed deep learning methods. It will allow for a significant acceleration of cell counting work in laboratories and a reduction of the burden on staff. Doing this work by using a computer will also reduce human error and increase the accuracy and reduce the likelihood of mistakes. To achieve this goal, our work was related to the development of methods that can automatically count blood cells. We proposed an approach that employs RetinaNet based on CNN architecture to detect all three types of blood cells, i.e., RBCs, WBCs, and platelets simultaneously.

The main contribution of this work includes several points. We prepared our own training dataset and manually marked RBCs, WBCs and platelets in the images. Then, we adapted and trained RetinaNet to recognize three types of cells simultaneously by presenting a wide collection of microscopic medical images. Next, we prepared an application that counted cells recognized by the RetinaNet network. Then, we evaluated the impact of learning epochs and confidence thresholds on the performance and effectiveness of cell detection and counting for each class on several images by comparing the number of cells counted by the application with the manually counted number of correctly classified cells, incorrectly classified cells, and unclassified cells. Based on those preliminary results, we selected and tested two of the trained models to evaluate how accurately they mark RBCs, WBCs, and platelets with a bigger test set for subsequent confidence thresholds. Finally, we calculated the accuracy, precision, recall, and F1-score of automatic counting for each type of cells, determined the optimal confidence thresholds for each type of cells, and compared them with the state-of-the-art.

## 2. Materials and Methods

### 2.1. General Concept of CNN Construction

Deep learning is a method that simulates the human brain structure. This method consists of a series of algorithms for finding a hierarchical representation of the input data based on the way that the human brain senses an important part of a sensory data set. It is a part of machine learning, which revolves around the algorithms responsible for modeling high-level abstraction, using many layers composed of nonlinear transformations. Due to their high efficiency, DNNs are nowadays the most popular group of deep learning algorithms.

In recent years, the unrestrainable increase of the data amount has raised new challenges in machine learning in the area of scalability. It was particularly evident in the subject of object recognition and image processing. During the analysis of a small black and white image, each neuron of the hidden layer would still have to have thousands of weights. This fact causes problems of both computational and purely practical nature. Such problems are dealt with by the architecture of CNNs [25].

**Figure 1:**
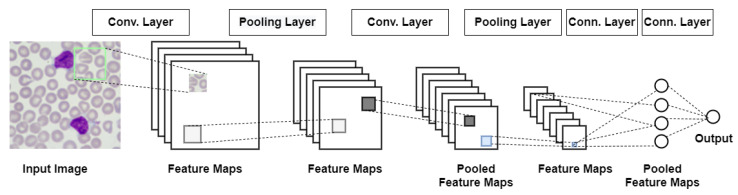
The sequence of transformations involved in the convolutional network for recognizing blood cells.

A CNN is a class of DNNs, most commonly applied to analyzing images and object recognition. Figure 1 shows the sequence of transformations involved in a typical convolutional network [26] that has been adopted in our research to recognize blood cells.

At first, the input image is scanned for feature selection. The checked rectangle is the filter that passes over the image. Activation maps are stacked atop another one for each of the employed filters. Secondly, the next rectangle is downsampled and the activation maps are downsampled. Next, a new set of activation maps is created by passing filters over the first downsampled stack. Then, the second set of activation maps is condensed by the second downsampling. Finally, the fully connected layer classifies the output with one label per node.

It is a solution taken from the human system of vision. Neurons are activated only when something is in the human field of vision, utilizing the fact that the features that represent only this small part of an image can relate to the entire surface of the image. Based on this knowledge, groups of neurons are created with common weights but located in different parts of the image. Several types of layers make CNN:

- Convolutional layers—they create feature maps based on systematically learned filters on input images and summarize the presence of these functions in the input. A map of the activity of a particular feature across the entire image area can be interpreted as a set of output signals from neurons of the same weight shall. The filter is a feature represented by one shared set of weights. The convolutional layer is operating in three dimensions, where instead of multiplying vectors, as in the classical approach, the convolution operation is applied and it gives better results when detecting a pattern [25,26];
- Pooling layers—they are used to streamline the computation. Combining the outputs of neuron clusters at one layer into a single neuron in the next layer by pooling layers reduces the dimensions of the data. Local pooling combines small clusters, and global pooling acts on all neurons of the convolutional layer. Pooling may calculate a maximum or an average. Max pooling uses the maximum value, and average pooling uses the average value from each of a cluster of neurons at the prior layer [25,26].
- Fully connected layer—uses the convolution results to classify the image into a label. The convolution output is flattened into a single vector of values representing the probability of belonging of a feature to that label. Each neuron receives weights that assign priority to the most appropriate label. Finally, neurons vote for each label, and the winner of this vote is the classification decision [26].

### 2.2. RetinaNet

RetinaNet is a one-stage detector that uses focal loss, whereby the lower loss is contributed by negative samples. The loss is concentrated in problematic samples, which improves the accuracy of prediction. With ResNet and Feature Pyramid Network (FPN) as the backbone for extraction of features and two task-specific subnetworks used for classification and bounding box regression, the formed RetinaNet achieves excellent performance and outperforms Faster R-CNN—the well-known two stage detector [27,28].

**Figure 2:**
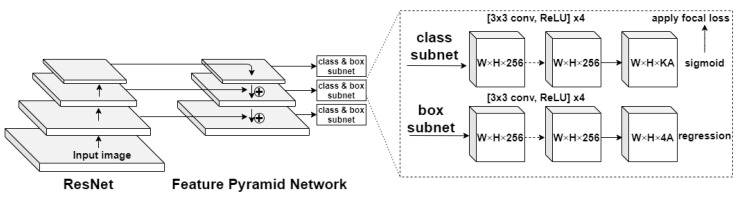
The architecture of RetinaNet Detector.

The architecture of RetinaNet shown in Figure 2 can be divided into three main groups [29]:

- a backbone FPN is used on the top of the ResNet model for constructing a rich multiscale feature pyramid from a single input image;
- a subnet used for classifying objects based on FPN outputs;
- a subnet that makes regression of the bounding box using the output data of the backbone network.

Feature pyramids are a basic component in recognition systems used for detecting objects at multiple scales. RetinaNet is based on the FPN presented in [30]. In a network containing residual blocks (ResNet), each layer feeds directly into the next layer and two to three jumped layers. In comparison, in traditional neural networks each layer feeds into the next layer. The training of a few layers can be skipped by using shortcut connections. It has been proven that training this type of network is easier than training in simple DNNs, and it particularly deals with the problem of accuracy degradation.

The fully convolutional nature of the network enables downloading an image of any scale and output proportional feature maps on multiple levels in the feature pyramid [31].

FPN consists of a bottom-up and top-down pathway. The bottom-up pathway is a convolutional network used for feature extraction, and the top-down pathway restores resolution to semantic information.

The classification and regression subnets are attached to each feature map obtained using FPN. The classification subnet predicts the object presence probability for each of the A anchors and K object classes at each spatial position. It applies four 3 × 3 convolutional layers, each with 256 filters and each followed by the Rectified Linear Unit (ReLU) activation, followed by a 3 × 3 convolutional layer with K × A filters. The regression subnet is identical to the classification subnet, except that 4A linear outputs are terminated per spatial location.

We used Keras implementation of RetinaNet object detection [32]. RetinaNet makes use of a ResNet-based backbone, from which a FPN is constructed. We used ResNet50 as the backbone. We took advantage of the possibility of using transfer learning. We set the weight option to the pretrained model when training and used the freeze backbone argument to freeze the backbone layers. We set the input batch size at 5 due to limitations in GPU memory. We trained the RetinaNet model with 36,382,957 parameters, which is equal to the number of trainable and non-trainable parameters.

### 2.3. Focal Loss

The imbalance between the background not containing objects and the foreground that holds interesting objects is the main issue for object detection model training. Focal loss is designed to assign greater weights to difficult, easily misclassified objects and downweight trivial ones. The goal is to minimize the expected value of the loss from the model and in the case of the cross-entropy loss, the expected loss is approximated as:

(1) $$\mathrm{CE}\left(p_{i},y\right)=-\log\left(p_{i}\right)\approx \frac{1}{n}\sum_{i=1}^{n}-\log p_{i}=\frac{1}{n}\sum_{i=1}^{n}L_{i}$$

where $L_{i}$ is the loss for one training example and the total loss *L* is approximated as the mean overall examples, $p_{i}\in \left[0,1\right]$ is the model’s estimated probability for the class y=1, and y∈{±1} specifies the ground-truth class [30].

The loss is calculated depending on the loss function definition. One of the most common loss functions is cross-entropy loss. This loss function is beneficial for image classification tasks, but different tasks need different loss functions. For example, in the detection problem in which bounding boxes are estimated around objects, a regression loss function can be used to get a measure of how well the bounding box is placed in the image.

The cross-entropy loss is used when the model contains the Softmax classifier. The Softmax classifier gives a probability score for each object class. The loss function is calculated as:

(2) $$L_{i}=-\log\left(\frac{e^{f_{y_{i}}}}{\sum_{j}e^{f_{j}}}\right)$$

where $L_{i}$ are all the training examples together, $f_{j}$ is the *j*-th element of the vector of class scores *f*, $y_{i}$ is the output for the correct class.

The Mean Square Error (MSE) is the most commonly used regression loss function. It can be computed as the squared norm of the difference between the true value and the predicted value:

(3) $$L_{i}={∥g-y_{i}∥}_{2}^{2}$$

where *g* are the predicted values and $y_{i}$ are the true ones. This loss function can be used when the goal is to find the coordinates of a bounding box when performing object detection.

### 2.4. Metrics

To quantitatively evaluate the results of cell counting, the following measures are defined.

The accuracy is defined by the following formula:

(4) $$\;\mathrm{Accuracy}\;=\frac{N_{\mathrm{expert}\;}\cap N_{\mathrm{count}\;}}{\max\left\{N_{\mathrm{expert}\;},N_{\mathrm{count}\;}\right\}}\cdot 100\%$$

where: $N_{expert}$—number of cells counted by an expert, $N_{count}$—cells counted by the application.

The classifier efficiency is evaluated based on its ability to correctly identify the number of cells belonging to one of the three classes. For each class, the quantitative measurement is performed based on True Positive (*TP*), False Positive (*FP*), True Negative (*TN*), and False Negative (*FN*) parameters.

Precision is the fraction of correctly identified samples of a given class to all correctly recognized samples. This value is given by the formula:

(5) $$\mathrm{Precision}=\frac{TP}{TP+FP}\cdot 100\%$$

Recall (sensitivity) is the number of correctly identified samples belonging to a given class to all samples belonging to that class. It is expressed by the formula:

(6) $$\mathrm{Recall}=\frac{TP}{TP+FN}\cdot 100\%$$

F1-score is the harmonic average of recall and precision, which can be expressed by the formula:

(7) $$F1-\mathrm{score}=\frac{2*\;\mathrm{Precision}\;*\;\mathrm{Recall}\;}{\;\mathrm{Precision}\;+\;\mathrm{Recall}\;}\cdot 100\%$$

## 3. Implementation of Cell Counting Algorithm

Our goal is to use an object detection and classification algorithm to detect and count three types of blood cells directly from a smear image. For this purpose, we have needed to train the RetinaNet network with selected settings and configurations based on training images with blood cell annotation. In this way, we created an application for recognizing and counting blood cells.

### 3.1. Datasets

For the learning and validation application, we used our own dataset consisting of 900 images containing WBCs, RBCs, and platelets. In the case of the validation dataset, we randomly selected 15 training images with annotations.

For application tests, we used images from the LISC dataset [33]. The dataset includes 251 images of resolution 720 × 576 acquired by a light microscope (Axioskope 40) with a magnification of 100×, recorded by a digital camera (Sony Model No. SSCDC50AP). From the test dataset, we randomly selected 131 images for counting WBCs, 64 images for counting platelets, and 15 images for counting RBCs. The different number of images selected for testing is due to the different number of individual cells in one image. Therefore, a small number of images for testing RBCs was selected, because of the large number of RBCs in individual images (average 121 RBCs per image). The situation is similar for the platelet count.

### 3.2. Image Labelling

**Figure 3:**
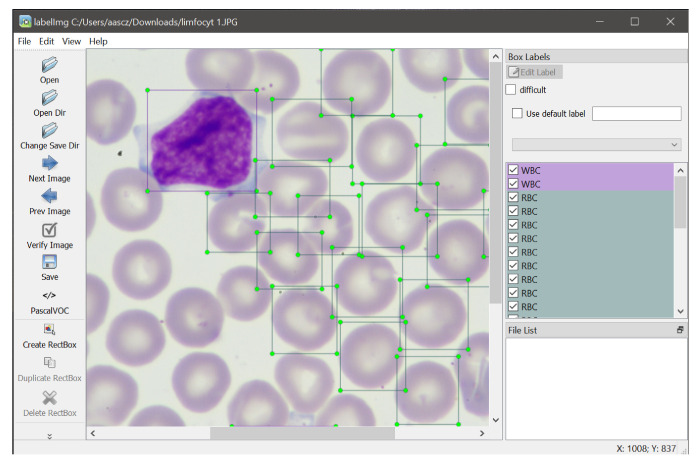
The process of marking the training dataset.

Before starting the network training process, we marked manually three types of cells in microscopic images using the LabelImg application, which is a graphical image annotation tool [34]. This process is shown in Figure 3. The objects in the images are divided into three categories: WBCs, RBCs, and platelets are marked accordingly. In this way, the annotations of blood cells were acquired for DNN training.

### 3.3. Training the RetinaNet of Object Recognition

We used the RetinaNet network with ResNet50 as the backbone with the input batch size set at 5, and the number of epochs set at 40 epochs, each for 500 steps for training. We used 900 images to train the network. The training process outputs a JSON file containing the network trained on these images, based on the set parameters.

**Figure 4:**
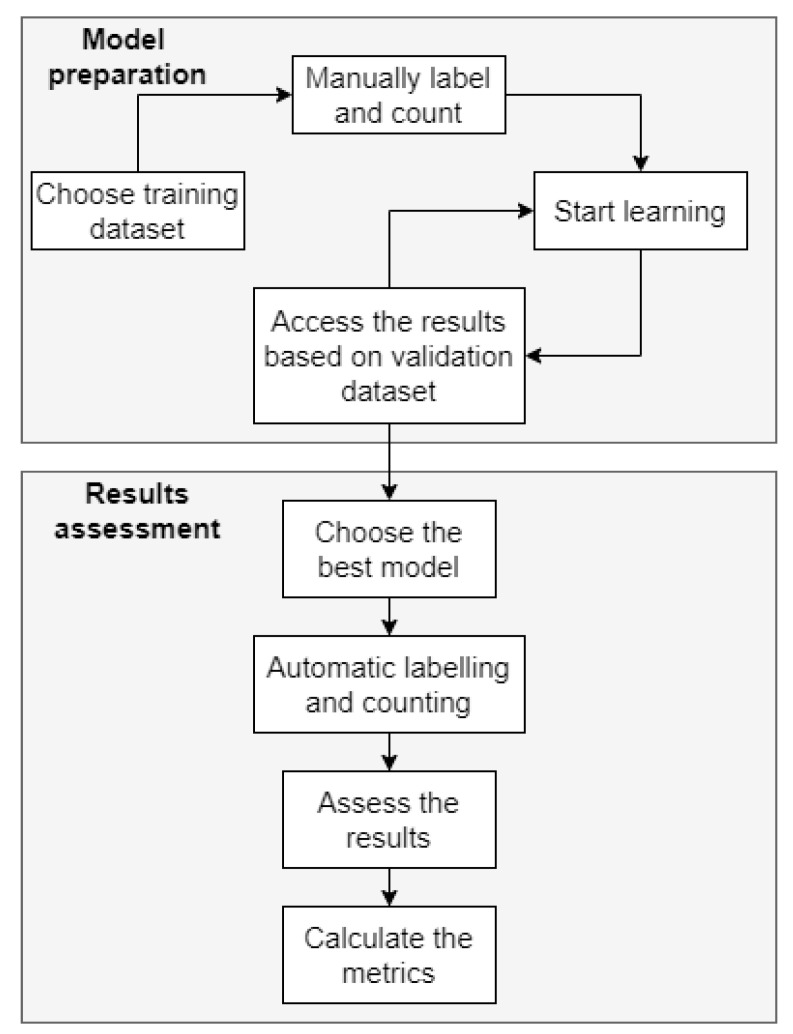
The cell counting workflow.

As a result of the training of each of the models, we obtained 40 files for each epoch. Then, we selected the results with 10, 15, 20, 25, 30, 35, and 40 epochs to investigate the impact of decreasing loss function on the detection accuracy. The workflow of the network learning process is presented in Figure 4.

**Figure 5:**
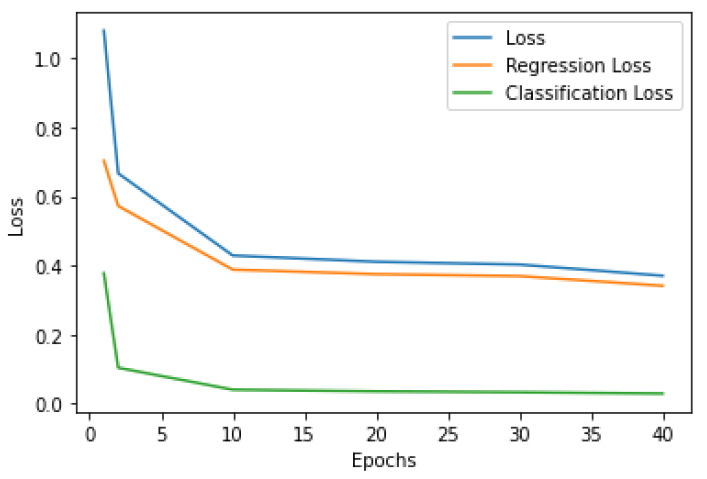
Learning curve of the RetinaNet blood cells identification (500 steps per epoch).

Figure 5 shows the learning curve of the RetinaNet algorithm to detect blood cells relative to the regression and classification loss function, as well as according to the sum of losses.

### 3.4. Selection of the Optimal Model

The criteria for selecting the best model variant were based on observation of the loss function, which decreased during learning from epoch to epoch. Additionally, we manually validated the results obtained after 10, 15, 20, 25, 30, 35, and 40 learning epochs using a validation set consisting of 15 images not used for training. We assessed the efficiency of blood cell counting by calculating the mean F1-score for each of the considered thresholds for each epoch. The results of the preliminary analysis are presented in Table 1, Table 2 and Table 3.

The additional aim of this validation was to compare the quality of cell counting after passing a certain number of epochs and to find the optimal model for further testing. The learning process was quite long. For a detailed analysis, we selected models trained with 10 and 30 epochs. The model trained with 10 epochs achieved very high F1-score results, and the loss function was stabilized for it. The model obtained after learning with 30 epochs achieved the highest F1-score values. We conducted research on a larger testing dataset for these two selected models and calculated metrics, such as F1-score, accuracy, precision and recall, allowing for an in-depth and comprehensive assessment of the quality of the RetinaNet model.

Analyzing the results of the F1-score presented in Table 1, Table 2 and Table 3, obtained on the basis of counting 3 types of cells in 15 images for each model and each threshold, it turned out that the best results of RBCs, WBCs, and platelet counting was achieved by RetinaNet trained during 30 epochs. That model returns the highest values of recognized RBCs, platelets, as well as WBCs. The same maximum values of F1-score values for WBCs also occur in other models, except the RN40. However, taking into account the maximum values of F1-score counting of all three types of cells from Table 1, Table 2 and Table 3, it can be indicated that the best is the RN30 model.

## 4. Experiments and Results

After the network training, we performed tests using a specially developed application, which allows for the import of trained models, cell detection, and presentation of results in a graphical and numerical form.

The tests of the developed models were performed for 15 images with RBCs, 151 images with WBCs, and 64 images with platelets. The output of the deep learning model is an image with an appropriate marking of the recognized samples. To verify the correctness of the obtained results, we counted all marked cells applied to their type in the dedicated application. Thus, our application counts different cells in the selected testing dataset with a different confidence threshold for selected models (RetinaNet model trained with 10 epochs (RN10) and the RetinaNet trained with 30 epochs (RN30)). We compared the results obtained for both types of models in order to check the impact of loss function values on the performance of object recognition.

It should be noted that the confidence threshold plays an instrumental role. Accuracy of identification and counting significantly depends on the appropriate confidence threshold setting. The values of different measures to estimate the accuracy of the recognition and counting of blood cells for testing data were presented in Table 4, Table 5, Table 6, Table 7, Table 8, Table 9 and Table 10.

**Figure 6:**
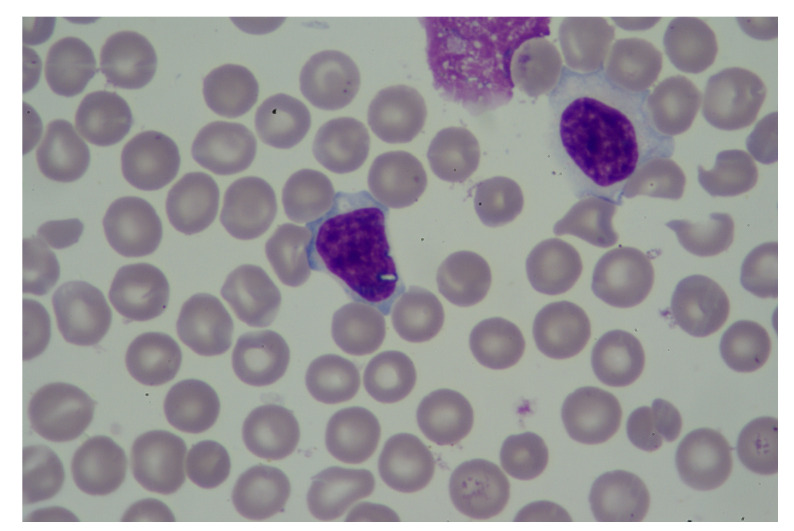
An example of blood smear image from the validation dataset.

**Figure 7:**
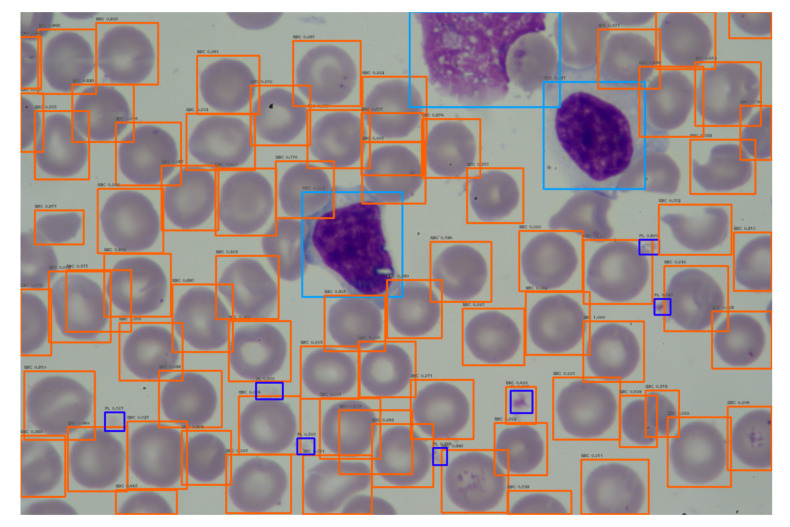
An example of blood smear image with recognized RBCs, WBC and platelets by the RN30 model.

To visualize the operation of the proposed labeling and blood cell counting method, we presented one of the images from the validation set. Figure 6 shows an original blood smear image, and Figure 7 shows the same image with automatically drawn bounding boxes, labels, and probabilities of each marked blood cell. It was returned by our application using the RN30 model with the confidence threshold of 0.35. Recognized cells were automatically marked on the bounding boxes according to their type. Orange bounding boxes mark RBCs, light blue mark WBCs, and dark blue mark platelets. It is seen in Figure 7 that WBCs and platelets are detected without error. Almost all RBCs are correctly labeled. However, three erroneous orange frames are also noticed, which includes a part of two neighboring RBC cells.

### 4.1. Results for the RN10

Table 4 contains the determined values of accepted quality measures for the RetinaNet model after 10 learning epochs (RN10) for 15 test images. Table 5 includes an assessment of WBCs counting quality in 131 images, and Table 6 contains the results of counting platelets in 64 images. Table 7 contains the ground truth of cells and cell numbers counted by our application for the confidence thresholds considered.

### 4.2. Results for the RN30

Table 8 contains the calculated values of the adopted quality measures for the RetinaNet model after 30 learning epochs (RN30) for 15 test images. Table 9 includes an assessment of the WBCs counting quality in 131 images, and Table 10 contains the results of counting platelets in 64 images. Total estimated numbers of cells of different types for different confidence threshold values are presented in Table 11.

As it is apparent from Table 8, in order to count RBCs, it is best to use the optimal threshold of 0.25. However, to count WBCs and platelets, the threshold is much higher (0.65 and 0.35 sequentially for Table 9 and Table 10). Thus, appropriate thresholds for each type of cells are selected as follows:

- RBCs—Confidence Threshold: 0.25,
- WBCs—Confidence Threshold: 0.65,
- Platelets—Confidence Threshold: 0.35.

For the RN10 model, the optimal confidence thresholds determined based on Table 4, Table 5 and Table 6 are as follows: 0.25 for RBCS, 0.60 for WBCs, and 0.30 for platelets. Thus, the thresholds in the counting of WBCs and platelets in the RN30 model are higher and increased as a result of learning the RN10 for another 20 epochs. Higher confidence thresholds give greater certainty of correct recognition of individual blood cells.

**Figure 8:**
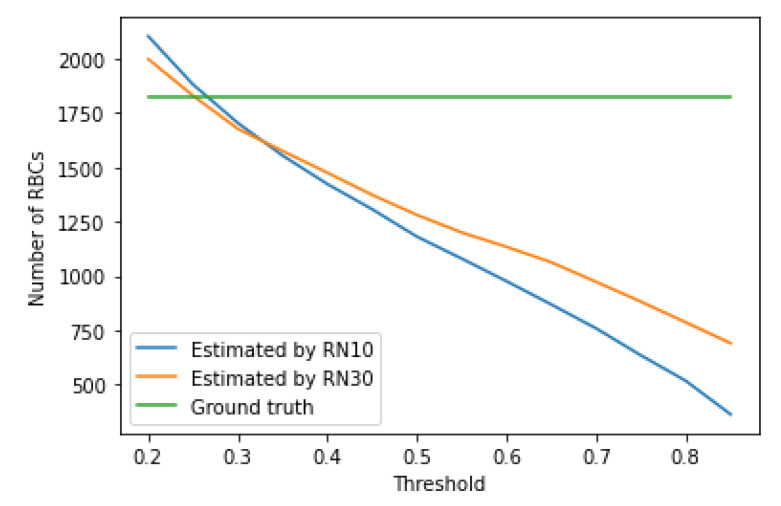
Number of detected RBCs vs. threshold value.

**Figure 9:**
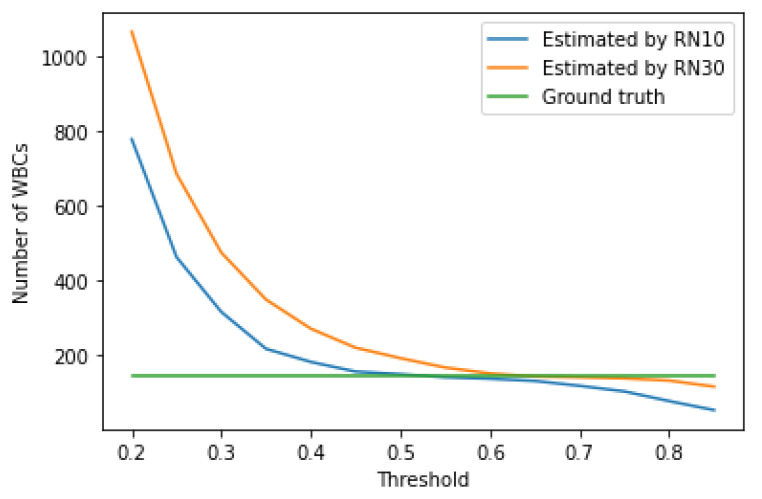
An example of blood smear image with recognized RBCs, WBC and platelets by the RN30 model.

**Figure 10:**
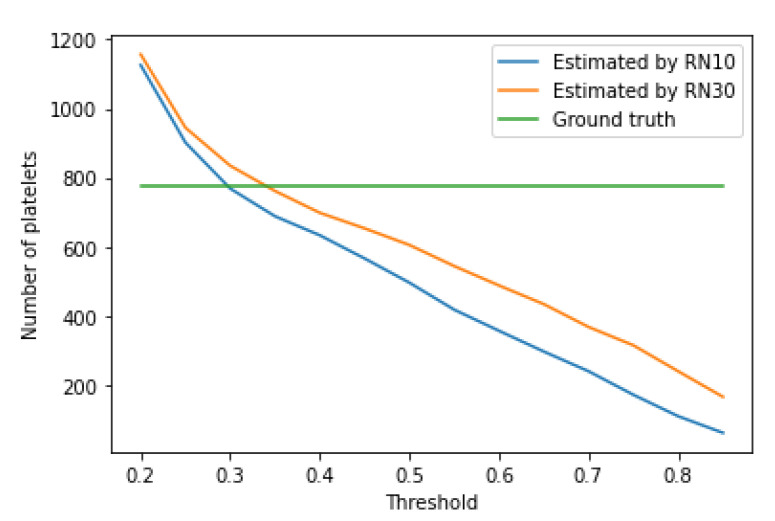
An example of blood smear image with recognized RBCs, WBC and platelets by the RN30 model.

The growth of the confidence threshold, which occurs in the RetinaNet model due to the network learning process, can be seen by analyzing and comparing Table 4, Table 5, Table 6, Table 7, Table 8, Table 9, Table 10 and Table 11, as well as analyzing Figure 8, Figure 9 and Figure 10, which shows the impact of the value of the confidence threshold on the number of counted cells concerning ground truths. From this figure, you can also effortlessly determine the optimal confidence thresholds for the three blood cell classes considered.

**Figure 11:**
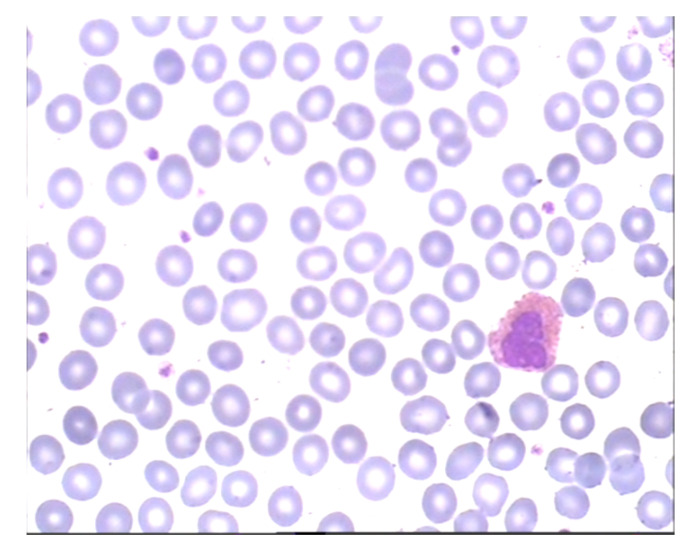
An example of blood smear image from the testing dataset.

**Figure 12:**
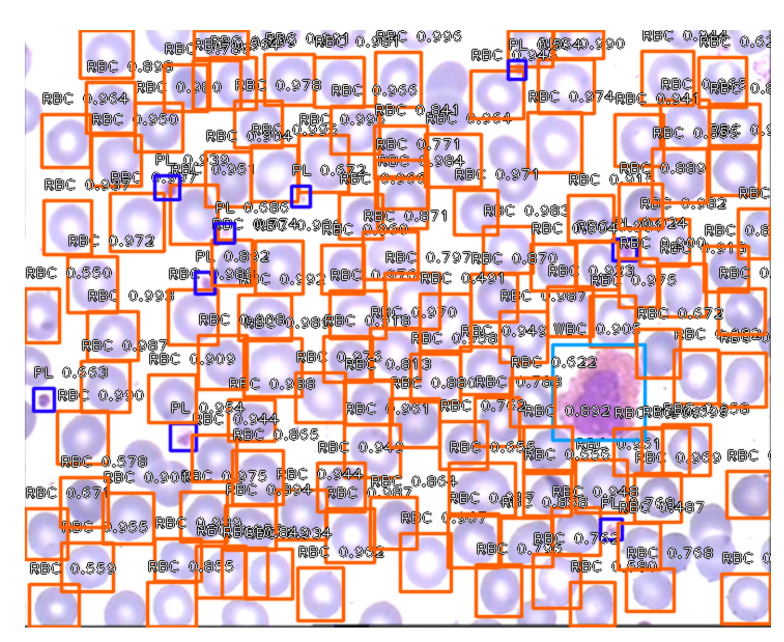
An example of blood smear image with recognized RBCs, WBC, and platelets by the RN30 model.

Figure 11 and shows the original image from the testing dataset. It was processed by our application, which automatically drew the bounding boxes, labels, and probabilities of each marked blood cell. An image processed using the RN30 model with the established confidence threshold of 0.45 is in Figure 12. Labels have determined colors, relevant names, and a probability value for each blood cell. At an established confidence threshold of 0.45, the WBC was correctly recognized and all platelets have been correctly recognized, labeled, and counted. The vast majority of RBCs are recognized and labeled correctly. At this confidence threshold, the RN30 model counts RBCs with an accuracy of about 75 %. It is seen in Figure 12 that there are only a few unchecked RBCs (typical for this threshold value), especially the RBCs that are overlapped or trimmed near the edge of the image. The application correctly recognized and counted one WBC. It also correctly recognized and counted 9 platelets and 123 RBCs when the ground truth is 135. The application also returns the probability values of each marked cell and the average probability of all recognized cells depending on their type. In this case, the average probability for WBCs was 0.905, for RBCs it was 0.875, and for platelets it was 0.784.

### 4.3. Comparison with the State-of-the-Art

To evaluate the performance of the proposed approach, we used the accuracy, precision, recall, and F1-score metrics, which are used most often for counting purposes. We compared the performance of the proposed approach with those obtained by other authors who dealt with the subject of cell counting for RBC, WBC, or platelet counting. It must be noted that only a few methods aimed to count both RBCs, WBCs, and platelets at the same time [24]. The selected methods work on the basis of deep learning as well as traditional image processing. Alam et al. [24] proposed an approach that employs YOLO to detect all three types of blood cells simultaneously. Their method does not require any greyscale conversion or binary segmentation and the whole process is fully automated. It is very similar to our approach because it uses deep neural networks to detect and count three types of cells. Dvanesh et al. [35] presented a method to digitally analyze the image of blood cells and find the RBC and WBC count values from the blood smear microscopic images using Digital Image Processing. Acevedo et al. [17] proposed a system for the automatic classification of peripheral blood cells (WBCs and platelets) by means of a transfer learning approach using convolutional neural networks. Di Ruberto et al. [36] proposed a system for detecting and quantifying red and white blood cells, which is based on the Edge Boxes method. That method is an approach for generating object bounding box proposals directly from edges.

A comparison of the RBC, WBC and platelet counting results with the results obtained by the other authors are reported in Table 12, Table 13 and Table 14. As can be observed, the proposed approach improves the counting performances; in particular, it significantly enhances accuracy. To highlight the performances obtained with the proposed method, in Table 12, we also report the number of images or ground truths used by the authors to test their approaches. The method proposed by [36] performed a higher precision, recall, and F1-score than our method.

The WBC counting results are reported in Table 13, which again have been directly compared with the results obtained by the other authors. The numerical results shown in Table 13 confirm the good performance of our approach, as it is able to detect WBCs with higher accuracy and precision than other methods. Only one method [36] performed higher recall and F1-score while losing accuracy and precision.

The platelet counting results are reported in Table 14, which have been compared with the results obtained by the same authors. The proposed approach obtained better accuracy than presented by Alam et al. [24] a and is slightly worse than presented by Acevedo et al. [17].

The obtained results are very satisfactory if we take into account that we are dealing with the recognition and counting of three types of cells simultaneously. However, it should be noted that similar to our approach was present only in one of the selected works [24] and in comparison to it, we obtained better performance in recognizing all three types of cells. Other works concerned the simultaneous recognition of two types of cells–WBCs and RBCs [35,36] or WBCs and platelets [17]. However, it should be noted that the compared results were obtained when tested on different datasets. For an accurate comparison of the results obtained, the approaches should be tested under the same conditions using the same datasets. Furthermore, the images used to test our approach contained average resolution images with a large number of cells (typically 100–150 cells per image) and the cells often overlapped each other, which impeded their correct recognition and counting.

## 5. Discussion

The results listed in Table 4, Table 5, Table 6, Table 7, Table 8, Table 9, Table 10 and Table 11 indicate that each cell type has its optimal confidence threshold. For optimal thresholds, the highest accuracy of recognizing and counting individual cells was obtained. Using one common confidence threshold generally cannot provide accurate results, because when choosing an indirect common threshold, for example, 0.55, all counting indicators will be worse and the application will not count exactly individual cells, as in the case of optimal confidence thresholds. Thus, each type of cells should be counted separately with the individually selected confidence threshold to obtain the most accurate results.

For the Retina model, after 30 epochs (RN30), at the confidence threshold of 0.25, the accuracy of RBCs counting by application is 99.7%, precision is 86.4%, and recall is 86.5%. Accuracy of WBCs counting by application at a confidence threshold of 0.65 is 98.6%, the counting precision is 97.9%, and recall is 96.5%. In the case of counting platelets, for the optimal threshold of 0.35, the accuracy is 97.8, precision is 92.8%, and recall is 90.8%. Almost all quality indicators for the RN10 model are slightly lower than for the RN30. Only in an assured range of confidence thresholds, the accuracy and precision of counting cells in the RN10 model is better than in the RN30. However, comparing F1-score values, the maximum value of this metric was obtained for the RN30 model.

In the light of the results presented above, general conclusions can be made. The model RN10 after the relatively short learning process (10 epochs) may quite accurately count the blood cells. Furthermore, learning up to 30 epochs improves almost all counting performance metrics, and it also grows the confidence threshold for the best results. The model trained by 40 epochs shows signs of overtraining visible on preliminary results for validation data. The presented results, however, partially present the complexity of the problem of counting blood cells. Regarding the selection of the optimal model for blood cell counting applications, we came to the conclusion that it is a difficult, complex, and time-consuming process because the accuracy of counting depends on the confidence threshold, the time of learning, the number of epochs, selection of performance evaluation metrics and perhaps many other factors which we did not include in this work. With such a wide study, the optimal confidence thresholds have been established, for which the application counted cells very accurately with high precision. We can dispose of redundant and incorrect estimates of the number of cells by selecting an appropriate confidence threshold for each cell type instead of a general threshold for all blood cells. The results obtained are very satisfactory for the recognition and counting of three cell types simultaneously compared to other works on cell counting, and we achieved better quality measure values for assessing the effectiveness of our approach for most of them.

Finally, we have to mention that a very big advantage of the application, in addition to the precise counting, is the appropriate marking of all recognized cells with labels and probabilities. It allows for easy verification of the obtained results. Marking recognized cells so far is still a rare functionality used in counting methods. Our method works in images of high resolution and dimensions. Different methods must divide a large image into a smaller one with a few number of cells in the individual image, which gives our method an additional advantage.

## 6. Conclusions

This article presents a machine learning approach to automatic identification and counting of blood cells from a smear image based on CNN RetinaNet. The proposed method is evaluated on the basis of publicly available datasets. The developed methods have been tested on different types of cells with different cell density in the images and they show promising results. The developed application returns the results in numerical and graphical form, which enables their simple verification. Additionally, the graphical results, i.e., labeled cells, ensure the probability of correct recognition of the right cell. We observed that in the case of the testing dataset, our method accurately recognizes and counts RBCs, WBCs, and platelets. However, the counting accuracy depends on the proper selection of the confidence threshold for individual cell classes.

An essential advantage is that the medical images do not require preliminary preparation, and all results are obtained after a single presentation of an image. All calculated metrics allow for in-depth and comprehensive evaluation of the quality of RetinaNet models. Due to the accuracy and performance of the detection, the proposed method has the potential to replace the manual identification of blood cells and the counting process. The developed application would allow for speeding up cell counting and increasing its accuracy.

## Figures and Tables

**Table 1:** Preliminary F1-scores for the recognition of RBCs, obtained from the analysis of 15 images, used to select the optimal model.

Threshold	RBCs F1-Score [%]						
	RN10	RN15	RN20	RN25	RN30	RN35	RN40
0.20	87.11	87.36	86.81	87.47	85.65	87.97	87.99
0.25	87.51	87.59	87.97	88.18	87.05	87.68	88.40
0.30	87.57	87.85	87.97	88.39	88.51	87.56	87.99
0.35	87.94	86.99	88.12	88.47	87.22	87.09	86.29
0.40	86.47	84.61	86.85	87.32	86.35	85.91	84.22
0.45	84.04	82.74	84.96	85.37	84.82	83.92	82.86
0.50	81.14	80.64	83.25	83.39	82.49	82.08	81.26
0.55	78.00	78.26	80.69	80.95	80.36	80.00	78.51
0.60	73.95	73.83	78.64	78.92	78.13	77.80	75.97
0.65	69.39	70.16	75.28	76.09	76.10	75.55	73.91
0.70	64.20	66.31	72.14	73.52	72.71	72.45	71.11
0.75	56.67	61.09	68.52	69.73	69.46	68.82	67.76
0.80	48.42	52.19	62.34	65.03	64.92	65.03	64.57
0.85	36.80	43.32	55.41	58.65	60.44	60.83	59.70

**Table 2:** Preliminary F1-score values for the recognition of WBCs, obtained from the analysis of 15 images, used to select the optimal model.

Threshold	WBCs F1-Score [%]						
	RN10	RN15	RN20	RN25	RN30	RN35	RN40
0.20	21.18	28.57	26.28	24.32	19.46	19.88	14.46
0.25	36.73	43.90	39.13	33.96	26.67	27.20	18.28
0.30	51.43	55.74	48.57	42.50	34.29	32.69	23.45
0.35	61.54	64.00	65.38	57.63	44.44	44.16	29.06
0.40	73.17	75.00	65.31	62.50	53.12	58.62	35.79
0.45	85.71	83.33	75.00	69.77	61.54	64.00	43.59
0.50	88.24	88.24	78.95	71.43	63.83	68.18	47.06
0.55	90.91	88.24	85.71	75.00	68.18	71.43	51.72
0.60	90.91	90.91	90.91	83.33	73.17	78.95	57.69
0.65	87.50	90.91	90.91	90.91	83.33	85.71	73.17
0.70	83.87	87.50	90.91	90.91	88.24	88.24	76.92
0.75	80.00	87.50	87.50	87.50	90.91	90.91	85.71
0.80	61.54	75.86	87.50	87.50	87.50	87.50	84.85
0.85	56.00	66.67	80.00	80.00	80.00	83.87	87.50

**Table 3:** Preliminary F1-score values for the recognition of platelets, obtained from the analysis of 15 images, used to select the optimal model.

Threshold	Platelets F1-Score [%]						
	RN10	RN15	RN20	RN25	RN30	RN35	RN40
0.20	73.95	71.07	75.62	75.23	73.17	73.49	66.95
0.25	80.65	81.72	82.58	81.82	81.23	80.75	73.35
0.30	83.99	85.46	83.03	83.57	86.53	80.94	76.88
0.35	82.05	85.36	83.91	85.45	85.11	82.39	80.60
0.40	80.13	81.70	82.39	83.60	83.97	81.97	80.51
0.45	77.82	80.00	80.41	80.95	81.19	80.41	79.21
0.50	73.84	78.08	77.03	78.32	78.50	77.35	77.51
0.55	68.42	73.76	75.27	74.82	75.00	73.45	74.47
0.60	58.30	68.66	64.59	68.18	70.85	67.18	68.66
0.65	47.83	58.06	57.61	60.80	64.59	60.24	63.53
0.70	41.28	52.14	52.77	52.77	55.00	53.16	53.78
0.75	35.24	42.73	38.32	44.84	49.57	47.58	49.35
0.80	27.86	34.45	30.39	35.24	41.28	39.07	39.81
0.85	13.98	24.37	24.37	24.37	27.86	26.13	26.13

**Table 4:** The accuracy, precision, recall, and F1-score of automatic counting of RBCs using RetinaNet model for 10 epochs (15 images).

Threshold	Accuracy [%]	Precision [%]	Recall [%]	F1-Score [%]
0.20	86.68	81.30	93.80	87.10
**0.25**	**96.91**	**87.23**	**90.01**	**88.60**
0.30	93.47	90.66	84.74	87.60
0.35	85.24	93.69	79.86	86.22
0.40	78.05	95.64	74.64	83.85
0.45	71.68	97.17	69.65	81.14
0.50	64.76	98.39	63.72	77.35
0.55	59.22	99.35	58.84	73.91
0.60	53.51	99.79	53.40	69.57
0.65	47.64	99.88	45.94	62.93
0.70	41.60	100.0	41.60	58.76
0.75	34.80	100.0	34.80	51.63
0.80	28.38	100.0	28.38	44.21
0.85	19.92	100.0	19.92	33.23

**Table 5:** The accuracy, precision, recall, and F1-score of automatic counting of WBCs using RetinaNet model for 10 epochs (131 images).

Threshold	Accuracy [%]	Precision [%]	Recall [%]	F1-Score [%]
0.20	18.51	18.25	98.61	30.80
0.25	31.17	30.74	98.61	46.86
0.30	45.71	45.08	98.61	61.87
0.35	66.67	64.81	97.22	77.78
0.40	79.56	77.35	97.22	86.15
0.45	92.90	89.68	97.20	93.29
0.50	97.30	93.24	96.50	94.85
0.55	97.22	96.43	93.75	95.07
**0.60**	**94.44**	**98.53**	**93.06**	**95.71**
0.65	90.28	98.46	90.14	94.12
0.70	81.25	99.15	81.69	89.58
0.75	70.83	99.02	72.14	83.47
0.80	52.78	97.37	52.86	68.52
0.85	36.11	100.0	37.14	54.17

**Table 6:** The accuracy, precision, recall, and F1-score of automatic counting of platelets using RetinaNet model for 10 epochs (64 images).

Threshold	Accuracy [%]	Precision [%]	Recall [%]	F1-Score [%]
0.20	69.18	68.56	98.97	81.01
0.25	86.36	82.93	95.90	88.94
**0.30**	**98.72**	**91.68**	**90.85**	**91.26**
0.35	88.45	96.08	86.09	90.81
0.40	81.39	97.48	79.84	87.78
0.45	72.79	99.47	73.06	84.24
0.50	63.80	99.80	64.50	78.36
0.55	53.79	100.0	54.49	70.54
0.60	45.96	100.0	46.86	63.81
0.65	38.25	100.0	39.11	56.23
0.70	30.94	100.0	31.54	47.96
0.75	22.21	100.0	22.21	36.34
0.80	14.25	100.0	14.25	24.94
0.85	8.09	100.0	8.09	14.96

**Table 7:** Ground truth and the estimated number of blood cells at different confidence thresholds for the RN10.

Threshold	RBC		WBC		Platelets	
	Ground truth	Estimated	Ground Truth	Estimated	Ground Truth	Estimated
0.20	1822	2102	144	778	779	1126
0.25	**1822**	**1880**	144	462	779	902
0.30	1822	1703	144	315	**779**	**769**
0.35	1822	1553	144	216	779	689
0.40	1822	1422	144	181	779	634
0.45	1822	1306	144	155	779	567
0.50	1822	1180	144	148	779	497
0.55	1822	1079	144	140	779	419
0.60	1822	975	**144**	**136**	779	358
0.65	1822	868	144	130	779	298
0.70	1822	758	144	117	779	241
0.75	1822	634	144	102	779	173
0.80	1822	517	144	76	779	111
0.85	1822	363	144	52	779	63

**Table 8:** The accuracy, precision, recall, and F1-score of automatic counting of RBCs using RetinaNet model for 30 epochs (15 images).

Threshold	Accuracy [%]	Precision [%]	Recall [%]	F1-Score [%]
0.20	91.24	81.18	88.41	84.64
**0.25**	**99.67**	**86.44**	**86.54**	**86.49**
0.30	91.99	90.51	83.35	86.78
0.35	86.39	93.20	80.60	86.45
0.40	80.90	94.91	76.87	84.94
0.45	75.30	96.43	72.69	82.89
0.50	70.25	97.11	68.30	80.19
0.55	65.81	97.75	64.40	77.64
0.60	62.18	98.23	61.15	75.38
0.65	58.29	98.59	57.53	72.66
0.70	53.35	99.18	52.97	69.05
0.75	48.35	99.66	48.08	64.86
0.80	43.14	99.87	43.02	60.14
0.85	37.87	100.0	37.91	54.98

**Table 9:** The accuracy, precision, recall, and F1-score of automatic counting of WBCs using RetinaNet model for 30 epochs (131 images).

Threshold	Accuracy [%]	Precision [%]	Recall [%]	F1-Score [%]
0.20	13.51	13.52	100.0	23.82
0.25	21.02	21.02	100.0	34.74
0.30	30.38	30.38	100.0	46.60
0.35	41.38	41.38	100.0	58.54
0.40	53.33	53.33	100.0	69.57
0.45	65.75	64.84	98.61	78.24
0.50	75.39	73.82	97.92	84.18
0.55	86.75	84.34	97.22	90.32
0.60	96.00	92.67	96.53	94.56
**0.65**	**98.61**	**97.89**	**96.53**	**97.20**
0.70	97.22	99.29	96.53	97.89
0.75	95.14	100.0	95.14	97.51
0.80	90.97	100.0	90.97	95.27
0.85	79.86	100.0	79.86	88.80

**Table 10:** The accuracy, precision, recall, and F1-score of automatic counting of platelets using RetinaNet model for 30 epochs (64 images).

Threshold	Accuracy [%]	Precision [%]	Recall [%]	F1-Score [%]
0.20	67.33	66.55	98.97	79.59
0.25	82.43	80.00	97.05	87.70
0.30	93.29	87.90	94.22	90.95
**0.35**	**97.82**	**92.78**	**90.76**	**91.76**
0.40	89.73	95.71	85.88	90.53
0.45	83.95	97.40	81.77	88.90
0.50	77.79	98.84	76.89	86.50
0.55	69.96	99.27	69.45	81.72
0.60	62.77	99.59	62.52	76.81
0.65	55.84	100.0	55.84	71.66
0.70	47.37	100.0	47.37	64.29
0.75	40.56	100.0	40.56	57.72
0.80	30.94	100.0	30.94	47.25
0.85	21.44	100.0	21.44	35.31

**Table 11:** Ground truth and the estimated number of blood cells at different confidence thresholds for the RN30.

Threshold	RBC		WBC		Platelets	
	Ground Truth	Estimated	Ground Truth	Estimated	Ground Truth	Estimated
0.20	1822	1997	144	1066	779	1157
0.25	**1822**	**1828**	144	685	779	945
0.30	1822	1676	144	474	779	835
0.35	1822	1574	144	348	**779**	**762**
0.40	1822	1474	144	270	779	699
0.45	1822	1372	144	219	779	654
0.50	1822	1280	144	191	779	606
0.55	1822	1199	144	166	779	545
0.60	1822	1133	144	150	779	489
0.65	1822	1062	**144**	**142**	779	435
0.70	1822	972	144	140	779	369
0.75	1822	881	144	137	779	316
0.80	1822	786	144	131	779	241
0.85	1822	690	144	115	779	167

**Table 12:** RBCs counting performance compared with the state-of-the-art.

	Alam [24]	Dvanesh [35]	Acevedo [17]	Ruberto [36]	Our Approach
Model	Tiny YOLO	ABCCS	-	Region proposal approach	RetinaNet50
No. images	60	63	-	108	15
Ground truths	792	-	-	-	1822
Accuracy	96.09	91.0	-	95.6	99.67
Precision	-	-	-	98.4	86.44
Recall	-	-	-	95.0	86.54
F1-score	-	-	-	96.6	86.49

**Table 13:** WBC counting performance compared with the state-of-the-art.

	Alam [24]	Dvanesh [35]	Acevedo [17]	Ruberto [36]	Our Approach
Model	Tiny YOLO	ABCCS	Vgg-16	Region proposal approach	RetinaNet50
No. images	60	63	1919	108	131
Ground truths	61	-	-	-	144
Accuracy	86.89	85.0	96.20	97.0	98.61
Precision	-	-	-	97.6	97.89
Recall	-	-	-	98.7	96.53
F1-score	-	-	-	98.0	97.20

**Table 14:** Platelet counting performance compared with the state-of-the-art.

	Alam [24]	Dvanesh [35]	Acevedo [17]	Ruberto [36]	Our Approach
Model	Tiny YOLO	-	Vgg-16	-	RetinaNet50
No. images	60	-	1919	-	64
Ground truths	55	-	-	-	144
Accuracy	96.36	-	99.61	-	97.82
Precision	-	-	-	-	92.78
Recall	-	-	-	-	90.76
F1-score	-	-	-	-	91.76

## Data Availability

Not applicable.

## Abbreviations

The following abbreviations are used in this manuscript:

CBC	Complete blood count
CNN	Convolutional neural network
DNN	Deep Neural Network
FN	False Negative
FP	False Positive
FPN	Feature Pyramid Network
MSE	Mean Square Error
RBC	Red Blood Cell
ReLU	Rectified Linear Unit
TN	True Negative
TP	True Positive
WBC	White Blood Cell
